# The combined effects of visual impairment, hearing loss, and olfactory dysfunction on cognitive impairment: An individually matched case–control study

**DOI:** 10.1002/alz.70439

**Published:** 2025-08-18

**Authors:** Jing Wu, Xiangjun Yin, Yang Liu, Weiqiang Ji, Jie Li, Gexin Xiao, Han Zhang, Ce Liu, Xiaomin Wu, Jingyi Xu, Shaohui Huang, Yue Hua, Feng Qiu, Ni Lin, Haotian Wu, Yaqiang Wang, Chengdong Xu, Qingfeng Du

**Affiliations:** ^1^ National Center for Chronic and Noncommunicable Disease Control and Prevention Chinese Center for Disease Control and Prevention Beijing China; ^2^ Guangdong Basic Research Center of Excellence for Integrated Traditional and Western Medicine for Qingzhi Diseases Guangzhou Guangdong China; ^3^ Hospital of Integrated Traditional Chinese and Western Medicine Southern Medical University Guangzhou Guangdong China; ^4^ School of Geography and Remote Sensing, Guangzhou University Guangzhou Guangdong China; ^5^ National Institute of Hospital Administration National Health Commission Beijing China; ^6^ State Key Laboratory of Resources and Environmental Information System Institute of Geographic Sciences and Natural Resources Research Chinese Academy of Sciences Beijing China; ^7^ School of Traditional Chinese Medicine Southern Medical University Guangzhou Guangdong China; ^8^ The Seventh Affiliated Hospital, Southern Medical University Foshan Guangdong China; ^9^ Guangdong Provincial Key Laboratory of Chinese Medicine Pharmaceutics Guangzhou Guangdong China

**Keywords:** case–control study, cognitive impairment, dementia, elderly, hearing loss, mild cognitive impairment, olfactory dysfunction, sensory dysfunction, subjective cognitive decline, visual impairment

## Abstract

**INTRODUCTION:**

The combined effects of visual impairment, hearing loss, and olfactory dysfunction on cognitive impairment remain unclear.

**METHODS:**

We conducted a case–control study with 25,432 adults aged 60 years or older. We used multiple logistic regression to analyze the effects of combined visual impairment, hearing loss, and olfactory dysfunction on cognitive impairment, while controlling for confounding factors.

**RESULTS:**

Single or combined visual impairment, hearing loss, and olfactory dysfunction negatively influenced cognitive impairment. Olfactory dysfunction had the greatest impact on dementia (odds ratio [OR] 2.37, 95% confidence interval 95% [CI] 2.01–2.79). For patients with combined sensory dysfunction, visual and olfactory dysfunction combined had the greatest impact on dementia (OR 6.50, 95% CI 3.78–11.20).

**DISCUSSION:**

Visual impairment, hearing loss, and olfactory dysfunction, as noninvasive functional biomarkers, provide a viable approach for the early detection and prevention of cognitive impairment, and can be utilized as effective means of early diagnosis and pre‐intervention of dementia.

**Highlights:**

- Olfactory dysfunction has a greater impact on cognition than visual impairment and hearing loss.
- The interaction of two sensory dysfunctions has a greater effect on cognition than one sensory dysfunction alone.
- Sensory dysfunction provides a viable approach for the early detection and prevention of cognitive impairment.

## 1 BACKGROUND

Cognitive impairment is a prevalent neurocognitive symptom among people over 60,^1^, ^2^, ^3^ of which dementia is the most pronounced phase. Subjective cognitive decline (SCD) refers to people who reported a decline of their own (subjective) cognitive function and is usually used as early clinical signs of dementia. Mild cognitive impairment (MCI) is a transitional phase between SCD and dementia. SCD, MCI, and dementia are generally recognized as clinical, diagnostic evidence of cognitive impairment.

Cognitive impairment causes a heavy burden of disease globally, especially in low‐ and middle‐income countries (LMICs).^4^ Dementia has been reported to be the seventh leading cause of death worldwide and was responsible for 1.6 million deaths in 2019, contributing to 25.3 million disability‐adjusted life‐years.^5^, ^6^ The annual global health spending on dementia has been projected to increase from $1.3 trillion per year globally in 2019 to $2.8 trillion by 2030.^7^ Since the 21st century, the rate of increase of dementia has slowed down in some high‐income countries such as the United States, the United Kingdom, and France, but remains high in LMICs.^8^

Given the irreversibility of the process leading to dementia and the lack of effective therapy, it is paramount to develop valid approaches for the early detection and prevention of dementia. Existing studies showed that early diagnosis and intervention can delay the development of MCI to dementia by 5 years.^9^ However, reliable, non‐invasive, easy‐to‐perform procedures for the early detection of dementia are still absent. Auditory, visual, and olfactory function are relatively easy to measure and provide potentially promising indicators for early detection of dementia. For example, existing studies suggest that hearing loss,^8^, ^10^ visual impairment,^10^ and olfactory dysfunction^11^, ^12^, ^13^, ^14^ can contribute to the development of dementia. However, there are inherent drawbacks to the existing study. First, the sample sizes for the combined effects of hearing, visual, and olfactory function on dementia were relatively small.^15^, ^16^, ^17^, ^18^, ^19^ Second, no large sample study has been conducted for LMICs concerning the effects of combined sensory dysfunction.^15^, ^16^, ^17^, ^18^, ^19^ Third, the effects of combined sensory dysfunction remain largely unclear, causing difficulty in evidence‐based, non‐invasive early detection of cognitive impairment in the elderly.

To mitigate the incidence of dementia in China, and to examine quantitatively how sensory dysfunctions influence dementia so that reliable, non‐invasive, and easy‐to‐perform procedures for the early detection of dementia can be developed, the Chinese Center for Disease Prevention and Control initiated the program “Prevention and Intervention on Neurodegenerative Disease for the Elderly in China (PINDEC).” In PINDEC, functions of visual, hearing, and olfactory were evaluated by onsite clinicians, and the degree of cognitive impairment of each participant was recorded. In total, 40,103 people over 60 years old in nine provinces across China were included. PINDEC is currently the largest community‐level survey dedicated to sensory dysfunction and cognitive impairment in China.

In this study, we quantify the contribution of visual impairment, hearing loss, and olfactory dysfunction and their combined effects on cognitive impairment using data from PINDEC. Our research fills the research gap on the impact of sensory dysfunction on cognitive function, thereby providing a practical method for the early detection and prevention of dementia at a large scale at the community level.

## 2 METHODS

### 2.1 Study design

An individually matched case–control study was conducted under the framework of PINDEC. From January 2015 to December 2021, cases were selected using a stratified random sampling method from 28 residential communities in nine provinces located in the eastern, central, and western regions of China to ensure that the cases selected were representative. In addition, cases in urban and rural areas were selected at a ratio of 1:1 to ensure that urban‐rural disparity is represented. Participants are those who have resided in the area for at least 12 months before the survey to minimize the confounding bias caused by migration. Those who refused to participate, with life‐threatening illnesses, hospitalized, or residing in senior care facilities were excluded.

**FIGURE 1 alz70439-fig-0001:**
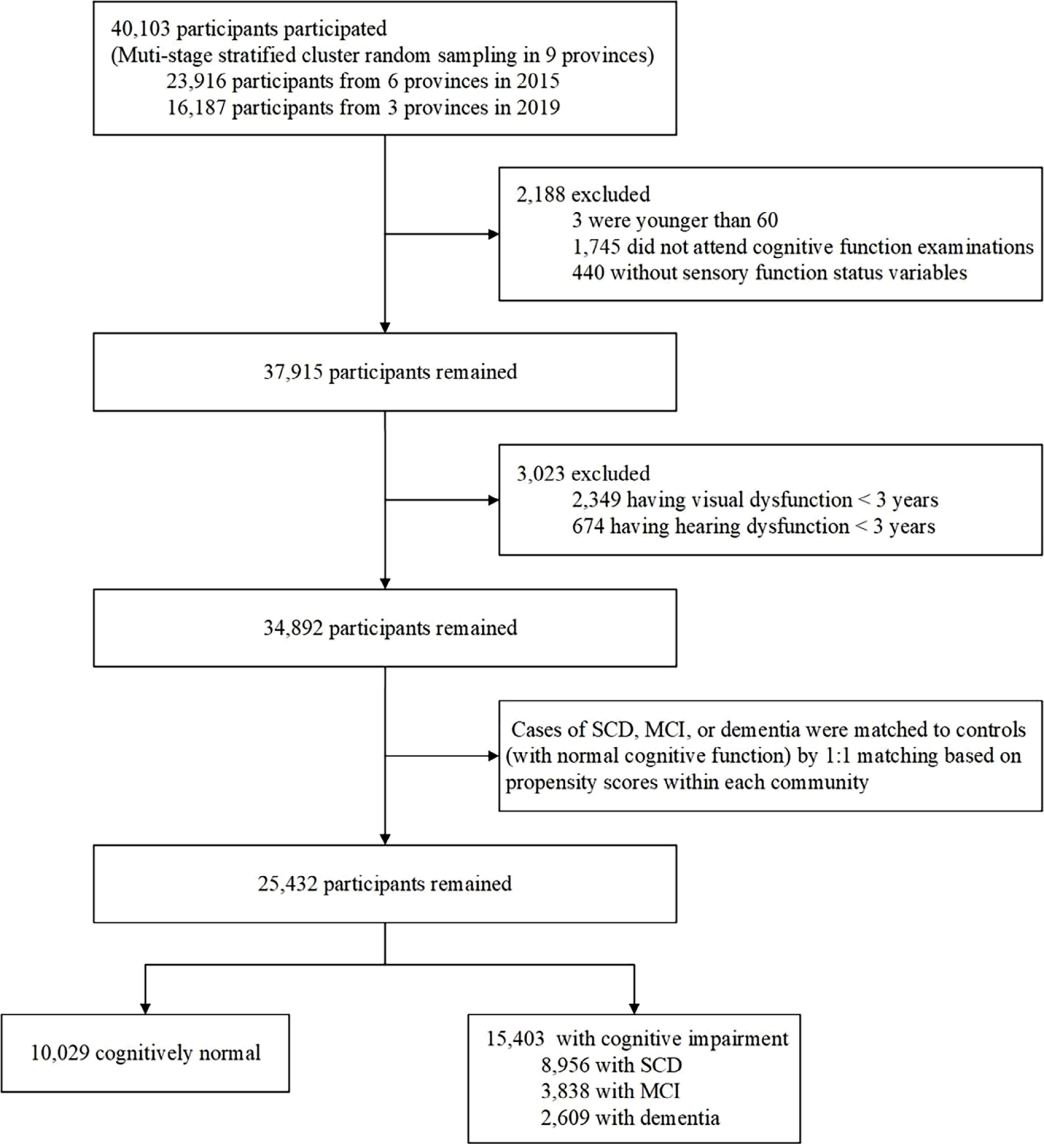
Study sampling scheme. MCI, mild cognitive impairment; SCD, subjective cognitive decline.

Forty thousand one hundred three cases were selected in total. After removing cases of having sensory dysfunction for less than 3 years, 34,892 participants remained. Then, the propensity score matching was performed, which included three key components: (1) each case was matched within 1 of 28 residential communities to control for local environmental and socioeconomic factors; (2) 1:1 nearest neighbor matching with replacement, each case was matched to a single nearest control (an individual with normal cognitive function) based on propensity scores, allowing controls to be matched to multiple cases. A caliper width of 0.02 standard deviations of the logit of the propensity score was applied to improve match quality; (3) The propensity score was estimated through logistic regression with cognitive impairment status as the dependent variable, adjusted for age and sex. Cases were excluded if they do not meet the eligibility criteria (Figure 1). After the propensity score matching process 25,432 participants remained.

After the elimination process, the cases consisted of individuals diagnosed with cognitive impairment, including SCD, MCI, and dementia. SCD was diagnosed if an individual has an Alzheimer's Disease 8 Questionnaire (AD8) score less than 2 and with a self‐reported memory or cognitive decline, but has not reached the DSM‐IV‐R criteria for dementia. MCI was diagnosed for those with an AD8 score of 2 or above, but did not reach the DSM‐IV‐R criteria for dementia. Dementia was diagnosed if one has reached the DSM‐IV‐R criteria for dementia. The control group consisted of individuals with normal cognitive function, that is, those who do not meet the DSM‐IV‐R criteria for dementia, having an AD8 score less than 2, and without self‐reported memory or cognitive decline.^20^, ^21^ For cases unable to communicate effectively, responses were collected through a proxy respondent who is familiar with the participant.

To ensure accuracy and reliability of the study, a strict data quality control and standardized procedures were carried out throughout the process. First, prior to the data collection, clinicians who will be conducting the survey were trained collectively on method of data collection and quality control criteria. Then, data were collected by the qualified clinicians using standardized questionnaires at the residence of the participants. All questionnaires were administered on tablets with automated skip patterns and logic checks. The survey covered demographic characteristics, physiological indicators, genetic factors, lifestyle behaviors, and other relevant information. Impairment of sensory function was diagnosed by the clinicians who conducted the survey, with reference to historical medical records. The whole process of the identification was audio‐recorded to ensure quality. After data collection, at least 5% of the audio recordings were randomly checked by external quality examiners to ensure quality. A dedicated national‐level project working group oversees the entire process at all selected residential communities across all provinces. All the above‐mentioned precautions and measures are taken to ensure the quality of the data and adherence to the established protocols.

The study project was approved by the Ethics Review Committee of the National Center for Chronic and Noncommunicable Disease Control and Prevention, the Chinese Center for Disease Control and Prevention (Batch No.: 201620; 201902), with all participants providing informed consent before enrollment.

**RESEARCH IN CONTEXT**

1. **Systematic review**: The authors reviewed the relevant literature. Some studies found an association between sensory dysfunction and cognitive impairment. Few studies with relatively large samples have investigated the prevalence of all three types of cognitive impairment (dementia, mild cognitive impairment [MCI], and subjective cognitive decline [SCD]) in association with three sensory dysfunctions (visual impairment, hearing loss, and olfactory dysfunction).
2. **Interpretation**: This study confirms the impairment of each sensory function has a significant effect on cognition, and found that interaction of combined sensory dysfunction showed greater effect on cognitive impairment. In terms of population‐attributable fractions (PAFs), olfactory dysfunction was higher than hearing loss and visual impairment.
3. **Future directions**: We suggest that visual impairment, hearing loss, and olfactory dysfunction examination be used as a non‐invasive early screening procedure for cognitive impairment in the community, to improve the screening efficiency of MCI in particular.

### 2.2 Measurement of exposure factors

The exposure factors in this study were sensory dysfunction, including visual impairment, hearing loss, and olfactory dysfunction. Visual impairment was defined as having a prior diagnosis of cataracts and glaucoma, resulting in visual impairment according to the Cataract[Fig alz70439-fig-0001] in the Adult Eye Preferred Practice Pattern of the American Association of Ophthalmology in 2022,^22^ and the European Glaucoma Society Terminology and Guidelines for Glaucoma 5th Edition.^23^ Hearing loss was defined according to the 2021 WHO World Hearing Report.^24^ Olfactory dysfunction was defined according to the International Consensus Statement on Allergy and Rhinology: Olfaction and the Expert Consensus on the Diagnosis and Treatment of Olfactory Dysfunction (2017) in China.^25^, ^26^ Diagnostics on‐site were checked for consistency with the historical records, any inconsistency was cross‐checked with the original data (Table 1). Participants having upper respiratory tract infections such as colds with recent infections were excluded.

**TABLE 1:** The methods and criteria used for diagnosing the sensory dysfunctions in the PINDEC survey.

Sensory dysfunction	Methods of assessment	Criteria used for on‐site diagnosis
Visual impairment	Historical diagnosis and on‐site clinical diagnosis.	Patients having cataract or glaucoma for three years or more.^23^, ^24^
Hearing loss		Patients with hearing loss (20 decibels or greater) for three years or more.^25^
Olfactory dysfunction		Patients experiencing olfactory dysfunction for 6 months or more.^26^, ^27^

Abbreviation: PINDEC, Prevention and Intervention on Neurodegenerative Disease for the Elderly in China.

Based on evidence from previous studies,^8^ the following covariates were included: demographic factors (gender, age, family history of dementia, marital status, occupation, education level), physiological factors (cerebrovascular disease, hypertension, obesity, diabetes, coronary heart disease, chronic obstructive pulmonary disease [COPD], chronic kidney disease, asthma, arthritis, cervical spondylosis, chronic insomnia, chronic constipation), behavioral factors (alcohol, smoking), and environmental factors (geographic region, residence, and PM_2.5_). For a detailed description of covariates, see Appendix Table S1.

### 2.3 Statistical analysis

Propensity score matching was used to minimize the influence of confounding factors.^27^ Binary logistic regression was employed to estimate the propensity score of participants with normal cognitive function to develop SCD, MCI, or dementia, using age and sex as independent variables. Each case of SCD, MCI, or dementia was individually matched to a control subject (those with normal cognitive function) by 1:1 nearest‐neighbor matching based on propensity scores, with a caliper of 0.02 (Tables S2–S4). A bilateral *p*‐value of 0·05 or smaller was considered to be statistically significant.

Multiple logistic regression was used to calculate the odds ratio (OR) and 95% confidence intervals (95% CIs) between sensory and cognitive function. The degree of cognitive function was divided into four types: normal function, SCD, MCI, and dementia, with the normal function as the baseline. The model was adjusted for all covariates, including demographic, genetic, physiological, and environmental factors. In addition, population‐attributable fractions (PAFs) were calculated based on the OR estimates to quantify the impact of each sensory dysfunction and their combinations on cognitive impairment.

Sensitivity analysis was conducted to ensure the robustness of the results. Specifically, sensory function was used as a single factor in regression analysis. Additional adjustments were made for factors such as low education levels, smoking, excessive alcohol consumption, cerebrovascular disease, high blood pressure, diabetes, obesity, and air pollution.

Statistical analysis was performed using SPSS, version 23.0. Propensity score matching was performed using the MatchIt package in R (version 4.3.1).

## 3 RESULTS

Among 25,432 participants included in the study, 15,403 were in the group with impaired cognitive function, and the rest (10,029) belonged to the control group. In the group with impaired cognitive function, 8956 had SCD, 3838 had MCI, and 2609 had dementia. Statistical analysis revealed that the percentages of manual workers, participants with lower education levels, and those who had cerebrovascular, chronic obstructive pulmonary, chronic insomnia, or chronic constipation disease were statistically significantly higher in the group with impaired cognitive function compared with the control group (*p*‐value of chi‐squared test < 0.05) (Table 2).

**TABLE 2:** Baseline characteristics of the study population.

	Controls	Cases			
Parameter	Cognitively normal (*n* = 10,029)	SCD (*n* = 8956)	MCI (*n* = 3838)	Dementia (*n* = 2609)	Total (*n* = 25,432)
**Demographic factors**					
Age (years)					
65+	7327 (73.1%)	6526 (72.9%)	2728 (71.1%)	2164 (82.9%)	18,745 (73.7%)
60∼ < 65	2702 (26.9%)	2430 (27.1%)	1110 (28.9%)	445 (17.1%)	6687 (26.3%)
Sex					
Female	5262 (52.5%)	4695 (52.4%)	2287 (59.6%)	1612 (61.8%)	13,856 (54.5%)
Male	4767 (47.5%)	4261 (47.6%)	1551 (40.4%)	997 (38.2%)	11,576 (45.5%)
Marital status					
Divorced, unmarried, or widowed	1933 (19.3%)	1696 (18.9%)	771 (20.1%)	733 (28.1%)	5133 (20.2%)
Married	8096 (80.7%)	7260 (81.1%)	3067 (79.9%)	1876 (71.9%)	20,299 (79.8%)
Occupation^a^					
Manual workers^*^	8096 (80.7%)	7136 (79.7%)	3250 (84.7%)	2381 (91.3%)	20,863 (82.0%)
Mental workers	1933 (19.3%)	1820 (20.3%)	588 (15.3%)	228 (8.7%)	4569 (18.0%)
Less education					
Yes^*^	6462 (64.4%)	5703 (63.7%)	2623 (68.3%)	2236 (85.7%)	17,024 (66.9%)
No	3567 (35.6%)	3253 (36.3%)	1215 (31.7%)	373 (14.3%)	8408 (33.1%)
**Genetic factors**					
Family history of dementia					
Yes	157 (1.6%)	196 (2.2%)	158 (4.1%)	81 (3.1%)	592 (2.3%)
No	9872 (98.4%)	8760 (97.8%)	3680 (95.9%)	2528 (96.9%)	24,840 (97.7%)
**Physiological factors**					
Cerebrovascular disease					
Yes^*^	663 (6.6%)	816 (9.1%)	591 (15.4%)	432 (16.6%)	2502 (9.8%)
No	9366 (93.4%)	8140 (90.9%)	3247 (84.6%)	2177 (83.4%)	22930 (90.2%)
Hypertension					
Yes	4438 (44.3%)	4166 (46.5%)	1650 (43.0%)	1349 (51.7%)	11,603 (45.6%)
No	5591 (55.7%)	4790 (53.5%)	2188 (57.0%)	1260 (48.3%)	13,829 (54.4%)
Obesity					
Yes	571 (5.7%)	476 (5.3%)	251 (6.5%)	155 (5.9%)	1453 (5.7%)
No	9458 (94.3%)	8480 (94.7%)	3587 (93.5%)	2454 (94.1%)	23,979 (94.3%)
Diabetes					
Yes	1449 (14.4%)	1450 (16.2%)	573 (14.9%)	444 (17.0%)	3916 (15.4%)
No	8580 (85.6%)	7506 (83.8%)	3265 (85.1%)	2165 (83.0%)	21,516 (84.6%)
Coronary heart disease					
Yes	927 (9.2%)	1044 (11.7%)	577 (15.0%)	351 (13.5%)	2899 (11.4%)
No	9102 (90.8%)	7912 (88.3%)	3261 (85.0%)	2258 (86.5%)	22,533 (88.6%)
Chronic obstructive pulmonary disease					
Yes^*^	690 (6.9%)	751 (8.4%)	511 (13.3%)	444 (17.0%)	2396 (9.4%)
No	9339 (93.1%)	8205 (91.6%)	3327 (86.7%)	2165 (83.0%)	23036 (90.6%)
Asthma					
Yes	243 (2.4%)	228 (2.5%)	183 (4.8%)	120 (4.6%)	774 (3.0%)
No	9786 (97.6%)	8728 (97.5%)	3655 (95.2%)	2489 (95.4%)	24,658 (97.0%)
Chronic kidney disease					
Yes	151 (1.5%)	183 (2.0%)	94 (2.4%)	71 (2.7%)	499 (2.0%)
No	9878 (98.5%)	8773 (98.0%)	3744 (97.6%)	2538 (97.3%)	24,933 (98.0%)
Arthritis					
Yes	645 (6.4%)	675 (7.5%)	275 (7.2%)	263 (10.1%)	1858 (7.3%)
No	9384 (93.6%)	8281 (92.5%)	3563 (92.8%)	2346 (89.9%)	23,574 (92.7%)
Cervical spondylosis					
Yes	1057 (10.5%)	1143 (12.8%)	616 (16.1%)	333 (12.8%)	3149 (12.4%)
No	8972 (89.5%)	7813 (87.2%)	3222 (83.9%)	2276 (87.2%)	22,283 (87.6%)
Chronic insomnia					
Yes^*^	2200 (21.9%)	2573 (28.7%)	1435 (37.4%)	1023 (39.2%)	7231 (28.4%)
No	7829 (78.1%)	6383 (71.3%)	2403 (62.6%)	1586 (60.8%)	18,201 (71.6%)
Chronic constipation					
Yes^*^	792 (7.9%)	908 (10.1%)	583 (15.2%)	524 (20.1%)	2807 (11.0%)
No	9237 (92.1%)	8048 (89.9%)	3255 (84.8%)	2085 (79.9%)	22,625 (89.0%)
**Behavioral factors**					
Alcohol					
Yes	2252 (22.5%)	1990 (22.2%)	721 (18.8%)	375 (14.4%)	5338 (21.0%)
No	7777 (77.5%)	6966 (77.8%)	3117 (81.2%)	2234 (85.6%)	20,094 (79.0%)
Smoking					
Yes	2162 (21.6%)	1914 (21.4%)	776 (20.2%)	387 (14.8%)	5239 (20.6%)
No	7867 (78.4%)	7042 (78.6%)	3062 (79.8%)	2222 (85.2%)	20,193 (79.4%)
**Environmental factors**					
Residence location					
Rural	4949 (49.3%)	4309 (48.1%)	2126 (55.4%)	1539 (59.0%)	12,923 (50.8%)
Urban	5080 (50.7%)	4647 (51.9%)	1712 (44.6%)	1070 (41.0%)	12,509 (49.2%)
Region					
Northern China	3354 (33.4%)	2770 (30.9%)	2235 (58.2%)	998 (38.3%)	9357 (36.8%)
Southern China	6675 (66.6%)	6186 (69.1%)	1603 (41.8%)	1611 (61.7%)	16,075 (63.2%)
PM_2.5_ ^b^					
Pollution	7328 (73.1%)	6485 (72.4%)	2769 (72.1%)	1789 (68.6%)	18,371 (72.2%)
Normal	2701 (26.9%)	2471 (27.6%)	1069 (27.9%)	820 (31.4%)	7061 (27.8%)

*Note*: Data are *n* (%).

Abbreviations: MCI, mild cognitive impairment; SCD, subjective cognitive decline.

^a^ Manual workers refer to farmers, fishermen, herders; mental workers refer to teachers, research or medical workers, office workers. The data of occupation do not include those whose occupations cannot be distinguished.

^b^ PM_2.5_ pollution level refers to the annual average concentration of PM_2.5_ is greater than 35.00 µg/m^3^; the normal level refers to the annual average concentration of PM_2.5_ ≤ 35.00 µg/m^3^.

^*^ The distribution of different groups of subjects in different cognitive status in the table was tested by Wilcoxon, *p* < 0.05.

The proportion of participants with single or compound sensory dysfunctions are shown in Table 3. Only visual impairment was most prevalent in cognitively normal, SCD and MCI case groups, while only olfactory dysfunction was the highest in the dementia group.

**TABLE 3:** Sensory function status of the study population by cognitive status.

	Controls	Cases			
Parameter	Cognitively normal (*n* = 10,029)	SCD (*n* = 8956)	MCI (*n* = 3838)	Dementia (*n* = 2609)	Total (*n* = 25,432)
**Sensory function status**					
Normal sensory function	8721 (87.0%)	7481 (83.5%)	2788 (72.6%)	1890 (72.4%)	20,880 (82.1%)
Only VI	516 (5.1%)	563 (6.3%)	362 (9.4%)	217 (8.3%)	1658 (6.5%)
Only HL	234 (2.3%)	272 (3.0%)	187 (4.9%)	93 (3.6%)	786 (3.1%)
Only OD	456 (4.5%)	470 (5.2%)	336 (8.8%)	296 (11.3%)	1558 (6.1%)
VH	65 (0.6%)	89 (1.0%)	84 (2.2%)	37 (1.4%)	275 (1.1%)
VO	21 (0.2%)	47 (0.5%)	37 (1.0%)	47 (1.8%)	152 (0.6%)
HO	11 (0.1%)	28 (0.3%)	24 (0.6%)	15 (0.6%)	78 (0.3%)
VHO	5 (0.0%)	6 (0.1%)	20 (0.5%)	14 (0.5%)	45 (0.2%)

*Notes*: Data are *n* (%). The distribution of cognitive status of people with different sensory functions in Table 3 was tested by Kruskal–Wallis test, *p* < 0.05.

Abbreviations: HL, hearing loss; HO, hearing loss and olfactory dysfunction; MCI, mild cognitive impairment; OD, olfactory dysfunction; SCD, subjective cognitive decline; VH, visual impairment and hearing loss; VHO, visual impairment, hearing loss, and olfactory dysfunction; VI, visual impairment; VO, visual impairment and olfactory dysfunction.

**FIGURE 2:**
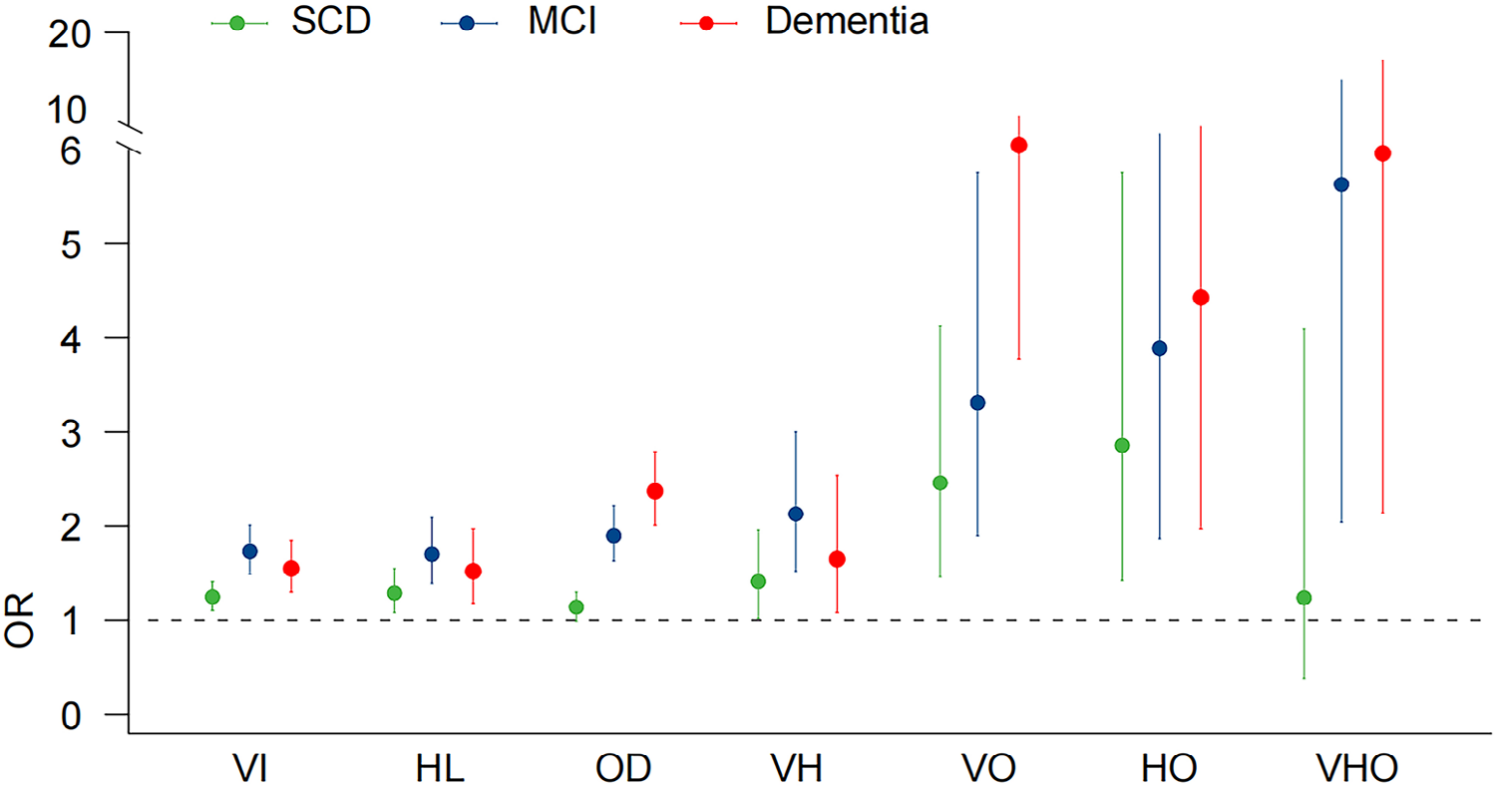
Association between sensory dysfunction and cognitive impairment. HL, hearing loss; HO, hearing loss and olfactory dysfunction; MCI, mild cognitive impairment; OD, olfactory dysfunction; OR, odds ratio; SCD, subjective cognitive decline; VI, visual impairment; VH, visual impairment and hearing loss; VHO, visual impairment, hearing loss, and olfactory dysfunction; VO, visual impairment and olfactory dysfunction.

The OR value of olfactory dysfunction was 2.37 (95% CI 2.01–2.79) for dementia, 1.90 (95% CI 1.63–2.22) for MCI, and 1.14 (95% CI 0.99–1.30) for SCD. The OR values for the effect of hearing loss was 1.70 (95% CI 1.39–2.09) for MCI, 1.52 (95% CI 1.18–1.97) for dementia and 1.29 (95% CI 1.08–1.55) for SCD. The OR value of visual impairment was 1.73 (95% CI 1.49–2.01) for MCI, 1.55 (95% CI 1.30–1.85) for dementia and 1.25 (95% CI 1.10–1.41) for SCD (Table 4 and Figure 2).

**TABLE 4:** Adjusted ORs and PAFs for sensory function status for SCD, MCI, and dementia.

	SCD		MCI		Dementia	
Parameter	OR (95%CI)	PAF (%)	OR (95%CI)	PAF (%)	OR (95%CI)	PAF (%)
**Sensory function status**						
Normal	1 (ref)	–	1 (ref)	–	1 (ref)	–
Only VI	1.25 (1.10–1.41)	1.58	1.73 (1.49–2.01)	4.54	1.55 (1.30–1.85)	3.46
Only HL	1.29 (1.08–1.55)	0.90	1.70 (1.39–2.09)	2.13	1.52 (1.18–1.97)	1.59
Only OD	1.14 (0.99–1.30)	0.83	1.90 (1.63–2.22)	5.23	2.37 (2.01–2.79)	7.75
VH	1.41 (1.02–1.96)	0.45	2.13 (1.52–3.00)	1.21	1.65 (1.08–2.54)	0.70
VO	2.46 (1.46–4.13)	0.87	3.31 (1.90–5.76)	1.36	6.50 (3.78–11.20)	3.18
HO	2.86 (1.42–5.76)	0.57	3.89 (1.87–8.11)	0.88	4.43 (1.97–9.98)	1.04
VHO	1.24 (0.38–4.10)	0.04	5.63 (2.04–15.57)	0.81	6.29 (2.14–18.43)	0.93

Abbreviations: HL, hearing loss; HO, hearing loss and olfactory dysfunction; MCI, mild cognitive impairment; OD, olfactory dysfunction; OR, odds ratio; PAF, population attributable fraction; SCD, subjective cognitive decline; VH, visual impairment and hearing loss; VHO, visual impairment, hearing loss, and olfactory dysfunction; VI, visual impairment; VO, visual impairment and olfactory dysfunction.

Olfactory dysfunction has the greatest impact on MCI and dementia among different sensory dysfunctions. The effect of single sensory dysfunction on dementia was reduced in the order of olfactory dysfunction (OR 2.37), visual impairment (OR 1.55), and hearing loss (OR 1.52). The impact of single sensory dysfunction on MCI was reduced in the order of olfactory dysfunction (OR 1.90), visual impairment (OR 1.73), and hearing loss (OR 1.70). The effect of single sensory dysfunction on SCD was reduced in the order of hearing loss (OR 1.29), visual impairment (OR 1.25), and olfactory dysfunction (OR 1.14).

Regarding the impact of the interaction of two sensory dysfunctions on cognition, visual impairment and olfactory dysfunction had the greatest impact on dementia (OR 6.50, 95% CI 3.78–11.20), and the impact decreased on MCI (OR 3.31, 95% CI 1.90–5.76), followed by SCD (OR 2.46, 95% CI 1.46–4.13). The effects of hearing loss and olfactory dysfunction on dementia (OR 4.43, 95% CI 1.97–9.98) and MCI (OR 3.89, 95% CI 1.87–8.11) were greater than those on SCD (OR 2.86, 95% CI 1.42–5.76). Visual impairment and hearing loss had a greater effect on MCI (OR 2.13, 95% CI 1.52–3.00) and dementia (OR 1.65, 95% CI 1.08–2.54) than that on SCD (OR 1.41, 95% CI 1.02–1.96) (Table 4 and Figure 2).

The effect of dual sensory dysfunction on dementia decreased in the order of visual impairment and olfactory dysfunction (OR 6.50), hearing loss and olfactory dysfunction (OR 4.43), and visual impairment and hearing loss (OR 1.65). The effect of two sensory dysfunctions on MCI decreased in the order of hearing loss and olfactory dysfunction (OR 3.89), visual impairment and olfactory dysfunction (OR 3.31), and visual impairment and hearing loss (OR 2.13). The following two sensory dysfunctions had a decreasing impact on SCD: hearing loss and olfactory dysfunction (OR 2.86), visual impairment and olfactory dysfunction (OR 2.46), and visual impairment and hearing loss (OR 1.41).

The PAFs of visual impairment for SCD, MCI, and dementia were 1.58%, 4.54%, and 3.46%, respectively. The PAFs of hearing loss for SCD, MCI, and dementia were 0.90%, 2.13%, and 1.59%, respectively. The PAFs of olfactory dysfunction for SCD, MCI, and dementia were 0.83%, 5.23%, and 7.75%, respectively. The total PAFs of visual impairment, hearing loss, and olfactory dysfunction for SCD, MCI, and dementia were 9.58%, 4.62%, and 13.81%, respectively (Table 4).

The results of sensitivity analysis showed that the results of model 1 and model 2 were similar to those of model 3, suggesting that the results of this study are reliable, and sensory dysfunction is an important risk factor for cognitive impairment. In model 1, the effects of sensory dysfunction on dementia were, in descending order, visual impairment and olfactory dysfunction (OR 10.33, 95% CI 6.16–17.32), hearing loss and olfactory dysfunction (OR 6.29, 95% CI 2.89–13.73), and olfactory dysfunction (OR 3.00, 95% CI 2.57–3.50), visual impairment and hearing loss (OR 2.63, 95% CI 1.75–3.95), visual impairment (OR 1.94, 95% CI 1.64–2.29), and hearing loss (OR 1.83, 95% CI 1.43–2.34). Likewise, in model 2, the effects of sensory dysfunction on dementia, in descending order, were visual impairment and olfactory dysfunction (OR 9.44, 95% CI 5.55–16.06), hearing loss and olfactory dysfunction (OR 6.26, 95% CI 2.82–13.90), and olfactory dysfunction (OR 2.96, 95% CI 2.53–3.48), visual impairment and hearing loss (OR 2.33, 95% CI 1.53–3.54), hearing loss (OR 1.76, 95% CI 1.37–2.27), and visual impairment (OR 1.72, 95% CI 1.45–2.05) (Table S5).

## 4 DISCUSSION

Our findings suggested that olfactory dysfunction had a greater impact on cognitive impairment than visual impairment and hearing loss. In addition, combined sensory dysfunction had a greater effect on cognitive impairment than any single sensory dysfunction alone, with the presence of all three sensory dysfunctions showing the most substantial impact on cognitive impairment. This study strengthens our understanding on the combined effect of sensory dysfunction on cognitive impairment and enriches insights into the 12 modifiable dementia risk factors highlighted in the Lancet report.^8^

The causal relationship between hearing and visual dysfunction and cognitive decline has been substantiated in existing studies,^10^ while only a few studies examine the association between olfactory dysfunction and cognitive impairment.^11^, ^13^ We have taken this issue into account in this study and examined the association between olfactory dysfunction and cognitive impairment by excluding 3023 participants who had visual or hearing impairment 2 years before developing olfactory dysfunction. This exclusion helps ensure that sensory dysfunction precedes cognitive decline, thus strengthening the causal inference. This study provides important evidence from a Chinese population on this topic.

Previous studies have reported that visual impairment, hearing loss, and olfactory dysfunction may contribute to cognitive impairment. For example, visual impairment and hearing loss can impair cognitive function, and cognitive function can be improved after cataract surgery or hearing aids. One prospective, longitudinal cohort study in the United States found that, based on 23,554 person‐years of follow‐up, cataract extraction was associated with significantly reduced risk (hazard ratio, 0.71) of dementia compared with patients without surgery.^28^ A multicenter, parallel‐group, unmasked, randomized controlled trial in the United States suggested that a hearing intervention might reduce cognitive change over 3 years in populations of older adults at increased risk for cognitive decline.^29^ Previous research has also shown that olfactory dysfunction can predict future cognitive decline in adults with normal cognitive function.^30^ Olfactory identification declined faster preceding dementia disorders, and Alzheimer's pathology may underlie these faster declines.^31^ In a cohort study including 7562 participants in the United States, functional vision and hearing impairment in older adults was associated with an increased risk of dementia.^32^ These studies suggested the association between single or combined sensory dysfunction and cognitive decline.

In this study, we found that olfactory dysfunction had a greater impact on cognitive impairment than visual impairment and hearing loss for MCI and dementia. The results could be attributed to distinct neuroanatomical pathway followed by olfactory system, which bypasses the thalamus and directly projecting to key regions implicated in Alzheimer's disease pathology, including the piriform cortex, amygdala, and entorhinal cortex (Figure S1).^33^ The neural circuits of olfactory perception and the brain regions associated with cognitive impairment exhibit a significant overlap and show notable correlations.^34^ Meanwhile, the potential mechanism of cognitive impairment caused by visual impairment, hearing loss, and olfactory dysfunction are mainly associated with cognitive reserve.^35^, ^36^

This study provides scientific basis and guideline for the prevention, screening, diagnosis, and early treatment of cognitive impairment. China currently has a low rate of early dementia diagnosis, at least 90% of people who develop dementia go undiagnosed at the early stage,^37^ which contradict with the goal set in “Healthy China Action (2019‐2030)” to curtail the growth rate of dementia.^38^ The relatively simple methodology of using sensory dysfunction as proxies for early signs of cognitive impairment provides a practical mean for large‐scale early diagnosis of dementia in low‐resource settings in LMICs such as China, and has the potential to be utilized by other LMICs as well as in developed countries with cost‐benefit considerations.

However, the screening procedure has not been a routine practice for early detection of cognitive impairment worldwide, especially the value of olfactory examination. We recommend prioritizing screening for people with olfactory dysfunction, once removed from exposure factor, dementia can be reduced by about 14.13% in this study. When people with hearing loss or visual impairment are screened, dementia can be reduced by about 8.39% and 7.60%, respectively.

This study also provides scientific evidence for. Specific interventions for visual impairment include cataract and glaucoma surgery, wearing hearing aids for hearing loss, and smell training interventions^39^ to improve olfactory function.

This study also suggested that health education should be promoted on the association between sensory dysfunction and cognitive impairment. Health promotions programs at community level should be promoted in China in order to reduce the growth rate of dementia by 2030.

Currently, the National Center for Chronic and Noncommunicable Disease Control and Prevention, China CDC has been coordinating the prevention and treatment of Alzheimer's disease in China from 2023 to 2025,^40^ utilizing the results in this study, and the local CDCs have been actively implementing the screening of sensory dysfunction as early signs of dementia. In addition, interventions are practiced to reduce the proportion of elderly people with early stages of cognitive impairment to develop into dementia.

The strengths of our study include a rigorous sampling design, standardized procedures, comprehensive quality control measures, large sample size, detailed covariate information, and robust statistical analysis methods. Shortcomings of this study include the potential for recall bias, a common issue in retrospective case–control studies. To mitigate this, we implemented a rigorously designed survey protocol to control recall bias effectively. In addition, the absence of detailed grading information on sensory dysfunction prevented a more detailed analysis of how varying degrees of sensory loss relate to cognitive impairment. Last but not least, the lack of comprehensive data on specific types of sensory dysfunctions and associated treatments (e.g., hearing aids) limited our ability to conduct subgroup analyses (Tables S6,S7).

## AUTHOR CONTRIBUTIONS

Jing Wu, Xiangjun Yin, and Qingfeng Du designed and supervised the study. Jing Wu and Qingfeng Du obtained funding for the study. Xiangjun Yin, and Han Zhang contributed to data collection and data quality control. Weiqiang Ji and Jingyi Xu did the statistical analysis and Chengdong Xu contributed to the methodology. Yang Liu, Weiqiang Ji, Jie Li, Ce Liu, Jingyi Xu, Xiaomin Wu, Haotian Wu, Yaqiang Wang, and Xiangjun Yin drafted the manuscript. Jing Wu, Xiangjun Yin, Qingfeng Du, and Chengdong Xu revised the manuscript. All authors attended the conference to discuss the content and approved the final version for submission.

## CONFLICT OF INTEREST STATEMENT

The authors declare no competing interests. Author disclosures are available in the supporting information. Author disclosures are available in Supporting information.

## CONSENT STATEMENT

All participants provided informed consent before enrollment.

## Supporting information



Supporting Information

Supporting Information

## Data Availability

The data analyzed during the current study are available from the corresponding author on reasonable request.
