# Supplementary material for: The combined effects of visual impairment, hearing loss, and olfactory dysfunction on cognitive impairment: An individually matched case–control study
Source: Alzheimers Dement. 2025 Aug 18;21(8):e70439. doi: 10.1002/alz.70439 (PMC12360912; doi:10.1002/alz.70439)
Supplement: Supplementary file 2 — Supporting Information [file ALZ-21-e70439-s001.docx]

**The combined effects of visual impairment, hearing loss, and olfactory dysfunction on cognitive impairment: An individually matched case-control study**

**Supplemental appendix**

[Table S1: Definition and diagnostic criteria for variables in this study 2](#_Toc193810418)

[Table S2: Comparison table of key variables before and after PSM matching between cognitively normal and SCD patients 6](#_Toc193810419)

[Table S3: Comparison table of key variables before and after PSM matching between cognitively normal and MCI patients 6](#_Toc193810420)

[Table S4: Comparison table of key variables before and after PSM matching between cognitively normal and dementia patients 7](#_Toc193810421)

[Table S5: Adjusted ORs for associations between sensory function status and SCD, MCI and dementia in model 1 and model 2 8](#_Toc193810422)

[Table S6: The Categories of olfactory function and their proportion distribution for different kinds of cognitive impairment patients 9](#_Toc193810423)

[Table S7: The proportion of patients whether with hearing loss wore hearing aids in different kinds of cognitive impairment patients 10](#_Toc193810424)

[Figure S1: Olfactory pathways 11](#_Toc193810425)

[References 12](#_Toc193810426)

Table S1: Definition and diagnostic criteria for variables in this study

| **Variables** | **Definition and diagnostic criteria** |
| --- | --- |
| Visual impairment | Visual impairment in this study were caused by cataract and glaucoma.   - A cataract is a degradation of the optical quality of the crystalline lens that affects vision. According to the 2022 American Ophthalmological Association Cataract in the Adult Eye Preferred Practice Pattern^1^, the diagnosis of cataract is mainly based on evaluation of visual impairment, ophthalmic valuation, supplemental ophthalmic testing including optical, corneal and macula testing and optic nerve and central nervous system testing. - According to European Glaucoma Society Terminology and Guidelines for Glaucoma, 5th Edition ^2^, the following tests are recommended at first assessment for glaucoma:  1. Visual acuity and refractive error 2. Slit lamp examination 3. Gonioscopy 4. Tonometry (strength of recommendation: strong) 5. Central corneal thickness (CCT) - Use of CCT-adjusted intraocular pressure (IOP) values is not recommended. 6. Visual field (VF) testing 7. Clinical assessment of the optic nerve head (ONH), retinal nerve fiber layer (RNFL) and macula. Binocular examination under pupil dilatation is preferable (except in angle closure). Optic disc and RNFL photography can be used. 8. Optical coherence tomography (OCT) of disc/RNFL/macula can be useful but the diagnosis of glaucoma cannot be made on the basis of OCT alone. |
|  |  |
| Hearing loss | A person is said to have hearing loss if their hearing capacity is reduced and they are not able to hear as well as someone with normal hearing. “Normal” hearing typically refers to hearing thresholds of 20 dB or better in both ears. Those with a hearing threshold above 20 dB may be considered “hard of hearing” or “deaf” depending upon the severity of their hearing loss.  According to hearing threshold in better hearing ear in decibels (dB) recommended by World Health Organization (WHO) in the world report on hearing^3^ in 2021, the following grades of hearing loss are recommended:   1. Normal hearing (less than 20 dB) 2. Mild hearing loss (20 to < 35 dB) 3. Moderate hearing loss (35 to < 50 dB) 4. Moderately severe hearing loss (50 to < 65 dB) 5. Severe hearing loss (65 to < 80 dB) 6. Profound hearing loss (80 to < 95 dB) 7. Complete or total hearing loss/deafness (95 dB or greater) |
| Olfactory dysfunction | Olfactory dysfunction refers to the abnormal odor perception caused by organic and / or functional lesions in each link of the olfactory pathway during the integration of odor perception, conduction and information analysis.   - Olfactory quantitative disorder:  1. Olfactory decline refers to the decline in the ability to sense, recognize, and distinguish smells. 2. Olfactory amnesia refers to the inability to perceive odors of any nature. 3. Olfactory allergy refers to the abnormal sensitivity to one or more odors.  - Olfactory characterization disorder:  1. Olfactory perversion refers to the distortion of the perception of odor properties. 2. Phantom smell refers to the illusory smell perception that occurs when there is no odor stimulation.   According to Chinese Expert Consensus on Diagnosis and Treatment of Olfactory Dysfunction (2017) ^4^, the diagnosis of olfactory disorders was mainly based on medical history, specialist examination, subjective evaluation (e.g. visual analogue scale, questionnaire of olfactory disorders) and psychophysical test (e.g. odor threshold test, odor discrimination test, odor discrimination test, odor identification test, T&T olfactometer test, Sniffin’ Sticks test, University of Pennsylvania smell identification test), and objective evaluation (e.g. event-related potentials, magnetic resonance imaging, functional magnetic resonance imaging, positron emission tomography-computerized tomography). |
| Less education | Less education referred to primary school level or below. |
| Hypertension | According to 2018 Chinese Guidelines for the Management of Hypertension ^5^, the following diagnosis criteria for hypertension are recommended:   - In the absence of antihypertensive agents, the clinic blood pressure was measured three times on different days, systolic blood pressure (SBP) ≥ 140mmHg and / or diastolic blood pressure (DBP) ≥ 90mmHg - SBP/DBP ≥140/90 mmHg in home blood pressure monitoring (HBPM) - Average 24-hour SBP/DBP ≥130/80 mmHg in ambulatory blood pressure monitoring (ABPM) - Average daytime SBP/DBP ≥135/85 mmHg in ambulatory blood pressure monitoring (ABPM) - Average nighttime SBP/DBP ≥120/70 mmHg in ambulatory blood pressure monitoring (ABPM) |
| Diabetes | According to Guideline for the Prevention and Treatment of Type 2 Diabetes Mellitus in China (2020 edition) ^6^, the following diagnosis criteria for diabetes are recommended:   - Typical symptoms of diabetes and random venous plasma glucose ≥11.1 mmol/L - Typical symptoms of diabetes and fasting venous plasma glucose ≥7.0 mmol/L - Typical symptoms of diabetes and oral glucose tolerance test (OGTT) 2 hour venous plasma glucose ≥11.1 mmol/L - Typical symptoms of diabetes and glycosylated hemoglobin (HbA1c) ≥6.5 mmol/L - Patients without typical symptoms of diabetes need to be re-examined to confirm. |
| Cerebrovascular disease | Cerebrovascular disease referred to stroke caused by traumatic brain injury (TBI) or other causes of stroke, and the diagnosis criteria was based on Chinese Guideline for primary care of ischemic stroke (2021)^7^ and Chinese guidelines for diagnosis and treatment of acute intracerebral hemorrhage 2019. ^8^ |
| Obesity | Obesity was diagnosed according to body mass index (BMI) ≥30 recommended by WHO.^9^ |
| Coronary heart disease | According to 2019 ESC Guidelines for the diagnosis and management of chronic coronary syndromes ^10^, six clinical scenarios most frequently encountered in patients are identified:   1. patients with suspected CAD and ‘stable’ anginal symptoms, and/or dyspnoea; 2. patients with new onset of HF or LV dysfunction and suspected CAD; 3. asymptomatic and symptomatic patients with stabilized symptoms <1 year after an ACS or patients with recent revascularization; 4. asymptomatic and symptomatic patients >1 year after initial diagnosis or revascularization; 5. patients with angina and suspected vasospastic or microvascular disease; 6. asymptomatic subjects in whom CAD is detected at screening. |
| Chronic obstructive pulmonary disease | According to Global Strategy for the Diagnosis, Management, and Prevention of Chronic Obstructive Lung Disease: the GOLD science committee report 2019. |
| Asthma | According to Key recommendations for primary care from the 2022 Global Initiative for Asthma (GINA) update. |
| Chronic kidney disease | According to Chronic kidney disease: assessment and management. London: National Institute for Health and Care Excellence (NICE); 2021. |
| Arthritis | According to Osteoarthritis in over 16s: diagnosis and management. London: National Institute for Health and Care Excellence (NICE); 2022. |
| Cervical spondylosis | According to Expert Consensus on the Diagnosis and Treatment of Cervical Spondylosis with Integrated Traditional Chinese and Western Medicine 2023. |
| Chronic insomnia | According to ‘Chinese chronic insomnia chronic disease management guidelines for the elderly 2023’. |
| Chronic constipation | According to ‘2020 European society of neurogastroenterology and motility guidelines on functional constipation in adults’. |
| Alcohol | Alcohol referred to that drinking alcohol > 210ml per week, which was based on the frequency of drinking. |
| Smoking | Smoking referred to current or previous smoking. |
| Tea drinking | Tea drinking referred to often drinking black tea or green tea. |
| Residence location | Residence location included rural or urban areas. |
| Region | Region in this study included three provinces (Liaoning, Beijing and Henan) in northern China and six provinces (Hubei, Shanghai, Yunnan, Sichuan, Guangxi and Guangdong) in southern China. |
| PM_2.5_ pollution | According to WHO global air quality guidelines.^11^ Particulate matter (PM_2.5_ and PM_10_), ozone, nitrogen dioxide, sulfur dioxide and carbon monoxide in 2021, PM_2.5_ pollution in this study refers to the annual average concentration of PM_2.5_ > 35ug / m^3^. |

Each case of SCD, MCI, or dementia was individually matched to a control subject (those with normal cognitive function) by 1:1 nearest-neighbor matching based on propensity scores, with a caliper of 0.02. The comparison table of key variables before and after matching is shown below. The tables reveal significant baseline disparities between case and control groups in age, gender, and geographic distribution prior to matching (p<0.001). Post-matching analyses demonstrated successful covariate balance across all matched pairs (p>0.05), indicating adequate comparability for subsequent analyses. All multivariate regression models were constructed using these matched datasets, incorporating appropriate adjustments for residual confounding factors.

Table S2: Comparison of key variables before and after PSM matching between participants with normal cognitive function and those with SCD

|  | **Unmatched** | | |  | **Propensity-score matched** | | |
| --- | --- | --- | --- | --- | --- | --- | --- |
|  | **Normal(n=14649)** | **SCD(n=11491)** | **p value** |  | **Normal(n=8956)** | **SCD(n=8956)** | **p value** |
| **Age** (years, median and IQR) | 70 (65-74) | 71 (66-75) | <0.001 |  | 70 (65-74) | 70 (65-74) | 0.708 |
| **Gender** (%) |  |  | <0.001 |  |  |  | 1 |
| Male | 7218 (49.2%) | 5328 (46.3%) |  |  | 4261 (47.5%) | 4261 (47.5%) |  |
| Female | 7431 (50.8%) | 6163 (53.7%) |  |  | 4695 (52.5%) | 4695 (52.5%) |  |
| **Region** (%) |  |  | <0.001 |  |  |  | 1 |
| North | 3976 (27.1%) | 3588 (31.2%) |  |  | 2770 (30.9%) | 2770 (30.9%) |  |
| South | 10673 (72.9%) | 7903 (68.8%) |  |  | 6186 (55.3%) | 6186 (55.3%) |  |

Table S3: Comparison of key variables before and after PSM matching between participants with normal cognitive function and those with MCI

|  | **Unmatched** | | |  | **Propensity-score matched** | | |
| --- | --- | --- | --- | --- | --- | --- | --- |
|  | **Normal(n=14649)** | **MCI(n=5018)** | **p value** |  | **Normal(n=3838)** | **MCI(n=3838)** | **p value** |
| **Age** (years, median and IQR) | 70 (65-74) | 70 (65-74) | <0.001 |  | 69 (65-73) | 69 (65-73) | 0.645 |
| **Gender** (%) |  |  | <0.001 |  |  |  | 1 |
| Male | 7218 (49.2%) | 1871 (37.2%) |  |  | 1551 (40.4%) | 1551 (40.4%) |  |
| Female | 7431 (50.8%) | 3147 (62.8%) |  |  | 2287 (59.6%) | 2287 (59.6%) |  |
| **Region** (%) |  |  | <0.001 |  |  |  | 1 |
| North | 3976 (27.1%) | 3286 (65.5%) |  |  | 2235 (58.2%) | 2235 (58.2%) |  |
| South | 10673 (72.9%) | 1732 (34.5%) |  |  | 1603 (41.8%) | 1603 (41.8%) |  |

Table S4: Comparison of key variables before and after PSM matching between participants with normal cognitive function and those with dementia

|  | **Unmatched** | | |  | **Propensity-score matched** | | |
| --- | --- | --- | --- | --- | --- | --- | --- |
|  | **Normal(n=14649)** | **Dementia(n=3734)** | **p value** |  | **Normal(n=2609)** | **Dementia(n=2609)** | **p value** |
| **Age** (years, median and IQR) | 70 (65-74) | 75 (68-81) | <0.001 |  | 73 (67-78) | 73 (67-78) | 1 |
| **Gender** (%) |  |  | <0.001 |  |  |  | 1 |
| Male | 7218 (49.2%) | 1174 (31.4%) |  |  | 997 (38.2%) | 997 (38.2%) |  |
| Female | 7431 (50.8%) | 2560 (68.6%) |  |  | 1612 (61.8%) | 1612 (61.8%) |  |
| **Region** (%) |  |  | <0.001 |  |  |  | 1 |
| North | 3976 (27.1%) | 1518 (40.7%) |  |  | 998 (38.3%) | 998 (38.3%) |  |
| South | 10673 (72.9%) | 2216 (59.3%) |  |  | 1611 (61.7%) | 1611 (61.7%) |  |

Table S5: Adjusted ORs for associations between sensory function status and SCD, MCI and dementia in model 1 and model 2

|  | **Model 1** | | | **Model 2** | | |
| --- | --- | --- | --- | --- | --- | --- |
|  | **SCD** | **MCI** | **Dementia** | **SCD** | **MCI** | **Dementia** |
|  | **OR(95%CI)** | **OR(95%CI)** | **OR(95%CI)** | **OR(95%CI)** | **OR(95%CI)** | **OR(95%CI)** |
| **Sensory function status** | | | | | | |
| Normal | 1(ref) | 1(ref) | 1(ref) | 1(ref) | 1(ref) | 1(ref) |
| Only VI | 1.27 (1.12,1.44) | 2.19 (1.91,2.53) | 1.94 (1.64,2.29) | 1.27 (1.12,1.43) | 2.14 (1.85,2.46) | 1.72 (1.45,2.05) |
| Only HL | 1.36 (1.13,1.62) | 2.50 (2.05,3.04) | 1.83 (1.43,2.34) | 1.31 (1.10,1.57) | 2.28 (1.87,2.79) | 1.76 (1.37,2.27) |
| Only OD | 1.20 (1.05,1.37) | 2.30 (1.99,2.67) | 3.00 (2.57,3.50) | 1.21 (1.06,1.38) | 2.32 (2.00,2.69) | 2.96 (2.53,3.48) |
| VH | 1.60 (1.16,2.20) | 4.04 (2.92,5.60) | 2.63 (1.75,3.95) | 1.51 (1.09,2.09) | 3.52 (2.53,4.91) | 2.33 (1.53,3.54) |
| VO | 2.61 (1.56,4.37) | 5.51 (3.22,9.43) | 10.33 (6.16,17.32) | 2.58 (1.54,4.32) | 5.37 (3.13,9.23) | 9.44 (5.55,16.06) |
| HO | 2.97 (1.48,5.97) | 6.83 (3.34,13.95) | 6.29 (2.89,13.73) | 2.92 (1.45,5.88) | 6.51 (3.16,13.40) | 6.26 (2.82,13.90) |
| VHO | 1.40 (0.43,4.59) | 12.52 (4.69,33.39) | 12.92 (4.65,35.93) | 1.33 (0.40,4.36) | 10.50 (3.89,28.36) | 10.93 (3.84,31.17) |

Model 1 only used sensory function as a single factor. Model 2 was adjusted for age, sex, less education, smoking, alcohol, cerebrovascular disease, hypertension, diabetes, PM_2.5_ pollution and obesity. HL=hearing loss. HO=hearing loss and olfactory dysfunction. MCI=mild cognitive impairment. OD=olfactory dysfunction. OR=odds ratio. SCD=subjective cognitive decline. VH=visual impairment and hearing loss. VHO=visual impairment, hearing loss and olfactory dysfunction. VI=visual impairment. VO=visual impairment and olfactory dysfunction.

In the study, olfactory function can be divided into three conditions: normal olfaction, conductive olfactory dysfunction, and sensorineural olfactory dysfunction and 7,298 patients only in 2019-2021 had their olfactory function classified.  Based on the dataset of 2019, we found that the distribution of patients' causes is shown in the following table. Among patients with dementia, sensorineural olfactory dysfunction accounted for 83.95% of the total olfactory dysfunction. Therefore, our study indicated that it was mainly reflect the relevant impact results of sensorineural causes. The impact of a small number of conductive causes on the results.

Table S6: The categories of olfactory function and their proportion distribution for different kinds of cognitive impairment patients

| **Condition of olfactory function** | **Cognitively normal(n=2361)** | **SCD(n=2133)** | **MCI(n=2134)** | **Dementia(n=670)** | **Total(n=7298)** |
| --- | --- | --- | --- | --- | --- |
| **Normal olfactory function** | 2228(94.40%) | 1999(93.70%) | 1983(92.90%) | 589(87.90%) | 6799(93.20%) |
| **Conductive causes** | 20(0.80%) | 33(1.50%) | 21(1.00%) | 13(1.90%) | 87(1.20%) |
| **Sensorineural causes** | 113(4.80%) | 101(4.70%) | 130(6.10%) | 68(10.10%) | 412(5.60%) |

In our study data, only the datasets from 2019 to 2021 contain information on whether patients with hearing loss wore hearing aids. Based on this dataset, we conducted analysis for the distribution of hearing function and the use of hearing aids among different populations with cognitive impairments is shown in the following table. We found that the proportion of elderly people wearing hearing aids among hearing loss patients is approximately 14.28% for patients with dementia, about 10.49% for those with MCI, and around 8.05% for those with SCD, with all proportions were small. Therefore, we believe that the conclusions of the study mainly reflect the relationship between cognitive impairment and the population of hearing loss patients who do not wear hearing aids.

Table S7: The proportion of patients whether with hearing loss wore hearing aids in different kinds of cognitive impairment patients

| **Condition of hearing function** | **Cognitively normal(n=2361)** | **SCD(n=2133)** | **MCI(n=2132)** | **Dementia(n=669)** | **Total(n=7295)** |
| --- | --- | --- | --- | --- | --- |
| Normal hearing function | 2277(96.4%) | 2046(95.9%) | 1989(93.3%) | 620(92.7%) | 6932(95.0%) |
| Hearing loss with hearing aid usage | 9(0.4%) | 7(0.3%) | 15(0.7%) | 7(1.0%) | 38(0.5%) |
| Hearing loss without hearing aid usage | 75(3.2%) | 80(3.8%) | 128(6.0%) | 42(6.3%) | 325(4.5%) |


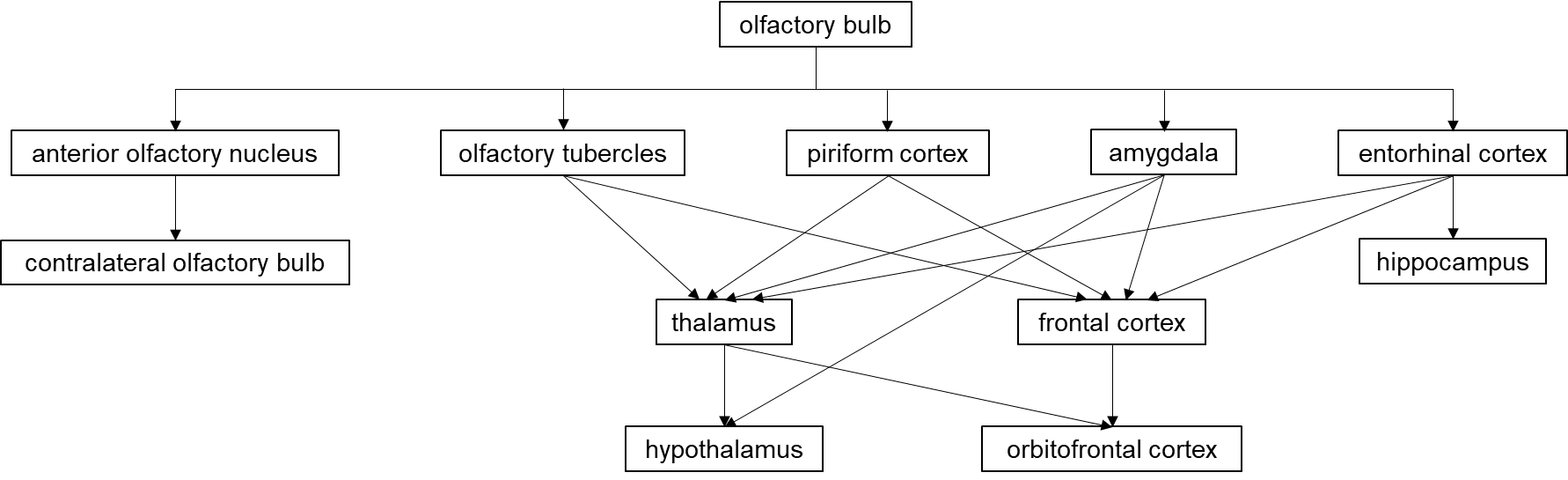


Figure S1: Olfactory pathways

**References**

1 Miller KM, Oetting TA, Tweeten JP, et al. Cataract in the Adult Eye Preferred Practice Pattern. *Ophthalmology* 2022; **129:** P1-126.

2 European Glaucoma Society Terminology and Guidelines for Glaucoma, 5th Edition. *Brit J Ophthalmol* 2021; **105:** 1-169.

3 World report on hearing. Geneva: *World Health Organization*, 2021.

4 Expert consensus on diagnosis and treatment of olfactory dysfunction ( 2017 ). *Chinese Journal of Otorhinolaryngology Head and Neck Surgery* 2018; **53:** 484-94.

5 2018 Chinese guidelines for the management of hypertension. *Chinese Journal of Cardiovascular Medicine* 2019**:** 24-56.

6 Guideline for the prevention and treatment of type 2 diabetes mellitus in China (2020 edition). *Chinese Journal of Diabetes Mellitus* 2021; **13:** 315-409.

7 Guideline for primary care of ischemic stroke (2021). *Chinese Journal of General Practitioners* 2021; **20:** 927-46.

8 Chinese guidelines for diagnosis and treatment of acute intracerebral hemorrhage 2019. *Chinese Journal of Neurology* 2019; **52:** 994-1005.

9 Obesity: preventing and managing the global epidemic. Report of a WHO consultation. *World Health Organ Tech Rep Ser* 2000; **894**: 1-253.

10 Knuuti J, Wijns W, Saraste A, et al. 2019 ESC Guidelines for the diagnosis and management of chronic coronary syndromes. *Eur Heart J* 2020; **41:** 407-77.

11 Singh D, Agusti A, Anzueto A, et al. Global Strategy for the Diagnosis, Management, and Prevention of Chronic Obstructive Lung Disease: the GOLD science committee report 2019. *Eur Respir J* 2019; **53**.

12 Levy ML, Bacharier LB, Bateman E, et al. Key recommendations for primary care from the 2022 Global Initiative for Asthma (GINA) update. *Npj Prim Care Resp M* 2023; **33:** 7.

13 Chronic kidney disease: assessment and management. London: *National Institute for Health and Care Excellence (NICE),* 2021.

14 Osteoarthritis in over 16s: diagnosis and management. London: *National Institute for Health and Care Excellence (NICE)*, 2022.

15 Xuejun C, Min Y. Expert Consensus on the Diagnosis and Treatment of Cervical Spondylosiswith Integrated Traditional Chinese and Western Medicine. *World Chinese Medicine* 2023; **18**.

16 Jingyu L, Wenjie X. Chronic insomnia chronic disease management guidelines for the elderly. *Research of Integrated Traditional Chinese and Western Medicine* 2023; **15**.

17 Serra J, Pohl D, Azpiroz F, et al. European society of neurogastroenterology and motility guidelines on functional constipation in adults. *Neurogastroent Motil* 2020; **32**: e13762.

18 WHO global air quality guidelines. Particulate matter (PM2.5 and PM10), ozone, nitrogen dioxide, sulfur dioxide and carbon monoxide. Geneva: *World Health Organization,* 2021.
